# Intravascular Residence Time Determination for the Cyanide Antidote Dimethyl Trisulfide in Rat by Using Liquid-Liquid Extraction Coupled with High Performance Liquid Chromatography

**DOI:** 10.1155/2016/6546475

**Published:** 2016-12-07

**Authors:** Deepthika De Silva, Steven Lee, Anna Duke, Siva Angalakurthi, Ching-En Chou, Afshin Ebrahimpour, David E. Thompson, Ilona Petrikovics

**Affiliations:** Department of Chemistry, Sam Houston State University, 1003 Bowers Blvd, Huntsville Texas, TX 77340, USA

## Abstract

These studies represent the first report on the intravascular residence time determinations for the cyanide antidote dimethyl trisulfide (DMTS) in a rat model by using high performance liquid chromatography coupled with ultraviolet absorption spectroscopy (HPLC-UV). The newly developed sample preparation included liquid-liquid extraction by cyclohexanone. The calibration curves showed a linear response for DMTS concentrations between 0.010 and 0.30 mg/mL with *R* ^2^ = 0.9994. The limit of detection for DMTS via this extraction method was 0.010 mg/mL, and the limit of quantitation was 0.034 mg/mL. Thus this calibration curve provided a tool for determining DMTS in the range between 0.04 and 0.30 mg/mL. Rats were given 20 mg/kg DMTS dose (in 15% Polysorbate 80) intravenously, and blood samples were taken 15, 60, 90, 120, and 240 min after DMTS injections. The data points were plotted as DMTS concentration in RBCs versus time, and the intravascular residence time was determined graphically. The results indicated a half-life of 36 min in a rat model, suggesting that the circulation time is long enough to provide a reasonable time interval for cyanide antagonism.

## 1. Introduction

Cyanide (CN) toxicity is caused by inhibiting the terminal oxidase of the mitochondrial respiratory pathway. This metabolic inhibition directs the cells into anaerobic metabolism, resulting in lactic acidosis [1]. One of the first symptoms of CN poisoning is deep and rapid ventilation caused by stimulation of carotid sinus. This is followed by the inhibition of cell respiration, leading to high oxygen levels in the venous blood [2]. The basic mechanism which removes CN from the body is its conversion to the less toxic compound of thiocyanate (SCN) in the presence of a sulfur donor [3]. This basic metabolic reaction serves as a basis for developing sulfur donor type CN antidotes. Presently, the two CN antidotes in the United States are Nithiodote™ and the Cyanokit®. Thiosulfate is the sulfur donor component of Nithiodote. Sodium nitrite is the other component and acts both as a methemoglobin former and as a nitric oxide former. Earlier studies were focusing on the methemoglobin forming effect of sodium nitrite as a scavenger type antidote [4]. Recent studies are focusing on the nitric oxide donor role within the mitochondria as the primary antidotal mechanism of sodium nitrite [5]. Cyanokit has the active ingredient of hydroxocobalamin, which can scavenge CN to form the stable cyanocobalamin complex [6].

New generation of therapies [7] (e.g., dimethyl trisulfide (DMTS) [8], cobinamide [9], and sulfanegen [10]) is developed to exploit the same paths of intervention as current therapies but does so more effectively. Additionally, formulations of these antagonists are being developed for intramuscular (IM) injection to enable first responders to dispense treatment more rapidly. This will be especially beneficial in remote locations and in events involving multiple victims.

DMTS, the promising next generation sulfur donor type cyanide antidote, is the simplest organic trisulfide that converts CN into the less toxic SCN [8, 11, 12]. It occurs naturally in garlic, onion, and other plants of the genus* Allium* [13]. DMTS is thus a natural part of the human diet. DMTS is formed by probiotics associated with yogurt and cheeses [14] and is an undesirable flavoring component of overly aged beers [15]. DMTS has been identified as a flavor determining factor in extruded potato snacks [16]. DMTS released from growing cabbages attracts moths whose larvae feed on the cabbages [17]; fermented Bermuda grass induces culex mosquitoes to lay eggs [18]; decaying vertebrate flesh attracts carrion beetles [19]; human urine provides a potential signal for locating victims trapped in collapsed buildings [20].

Here we report a development of sample preparation method that enables DMTS concentrations in blood to be determined by using high performance liquid chromatography coupled with ultraviolet absorption spectroscopy (HPLC-UV). Secondly, we report the intravascular residence time determination for DMTS (formulated with 15% Polysorbate 80 (Poly80)) in a rat model.

## 2. Experimental

### 2.1. Chemicals and Samples

All chemicals employed were of the highest purity commercially available and were used as received. Sodium chloride, HPLC grade water, cyclohexanone, and sodium heparin were purchased from J.T.Baker (PA, USA), HPLC grade acetonitrile was purchased from EMD Chemicals Inc. (NJ, USA), and Poly80 was purchased from Alfa Aesar (MA, USA). DMTS was purchased from SAFC (St Louis, MO) and dextrose from Sigma Aldrich (WI, USA). Screw cap vials (1.5 mL and 5 mL), 27 G × 13 mm and 25 G × 25 mm needles, 250, 100, and 50 *µ*L Hamilton Luer-lock syringes, and 1.7 mL microcentrifuge tubes were purchased from VWR International (Suwanee, GA, USA) and Polyspring inserts (100 *µ*L) from National Scientific (Rockwood, TN, USA). A 50 mg/mL lock solution was prepared by dissolving 1.25 g of sodium heparin in 25 mL of 5% w/v aqueous dextrose solution. A 0.9 w/v aqueous saline solution was prepared by dissolving 22.5 g of NaCl in 25 mL of distilled water. An 80 mg/mL heparin solution was prepared by dissolving 2.00 g of sodium heparin in 25 mL of distilled water. A DMTS stock solution (Poly80-DMTS) at a concentration of 50 mg/mL was prepared by dissolving DMTS in 15% Poly80 aqueous solution described by Kovacs et al. [21] and Petrikovics and Kovacs [12].

### 2.2. Instruments

The primary instruments used for the experiments were Varian ProStar HPLC (Model 210) with a UV detector (Model 340). A centrifuge (Galaxy 20R, VWR International, Suwanee, Georgia, USA), sonicator (Symphony™, VWR International), fixed speed mini vortex (VWR International, Suwanee, Georgia, USA), Heidolph Multi Reax mechanical shaker (Heidolph Instruments, Elk Grove Village, IL), micropipettes (Thermo Scientific, Waltham, MA), surgical dissection kit (Carolina Biological Supply Co., Burlington, NC), and a rat holder (Kent Scientific Corporation, Torrington, CT) were also used for these experiments. Precellys Lysing Kits having ceramic beads with average diameter of 1.4 mm were employed in conjunction with Precellys 24 tissue homogenizer (Bertin Technologies, Rockville, MD).

### 2.3. Animals

Male rats (250–300 g, Charles River Breeding Laboratories, Raleigh, NC) with catheters implanted on the jugular vein were housed at room temperature in a light controlled room (22 ± 2°C, 12 hrs light/dark cycle). The rats were furnished with water and Teklad Rodent Diet (W) 8604 (Teklad HSD, WI)* ad libitum*. Rats were handled in accordance with The Guide for the Care and Use of Laboratory Animals and accredited by American Association for the Assessment and Accreditation of Laboratory Animal Care (AAALAC) International. These experiments were approved by the Institutional Animal Care and Use Committee (IACUC) at Sam Houston State University (IACUC Permission: 15-09-14-1015-3-01).

### 2.4. HPLC Conditions Used Throughout the Study

A nonpolar silica based HPLC column with a Phenomenex Luna stationary phase consisting of bonded octane units was used (average pore size of 100 Å, average particle size of 5 *µ*m, and column dimensions 250 × 4.60 mm). The mobile phase was a 40 : 60 (V/V) blend of water and acetonitrile. The flow rate of the mobile phase was 1 mL/min and the column pressure was 100–105 bars. Injection volume of samples was 25 *µ*L. The UV detector monitored the absorbance at 215 nm wavelength. The peak areas were calculated automatically by Chromeleon 7 software.

### 2.5. Spiking Rat Blood with Poly80 Formulated DMTS and Partitioning Study

Aliquots of the DMTS stock solution were diluted with appropriate amounts of 15% (m/m) Poly80 to yield standard solutions having DMTS concentrations of 0.12, 0.36, 0.60, 0.84, 1.08, 2.40, and 3.60 mg/mL. A 100 *µ*L aliquot of the first standard solution was then added to 1100 *µ*L of intact rat blood in a heparinized microcentrifuge tube and was vigorously vortexed for 30 min. This process was repeated with each successive standard to obtain seven blood samples spiked, respectively, with DMTS at concentrations of 0.01, 0.03, 0.05, 0.07, 0.09, 0.20, and 0.30 mg/mL. This range was selected to correlate with blood concentrations following administration of highest expected doses of intravenous (residence time determination) DMTS. Each sample, after mixing with DMTS, was centrifuged (4°C, 13,500 rpm) for 10 min. The plasma portion was carefully removed using a micropipette. The sediment portion containing red blood cells (RBCs) was sonicated at 4°C for 10 min and vortexed for 30 seconds. The concentration of DMTS was measured in both plasma and RBCs for the partitioning study after extraction by cyclohexanone. For the calibration curve each spiked blood sample was extracted with cyclohexanone. The same sample preparation was applied for the spiked blood samples and the blood samples from the DMTS-treated rats.

### 2.6. DMTS Administration to Rat

For the intravascular residence time study, 20 mg/kg DMTS dose was applied intravenously by injecting the calculated volume of the Poly80-formulated DMTS solution (50 mg DMTS/15% Poly80). The following formula was used for determining the injection volume of the test solution in the rat model, where *X* is weight of the rat in units of grams; *Y* is sulfur donor (DMTS) dose in units of mg of sulfur donor (DMTS) per kg of rat; *Z* is concentration of sulfur donor (DMTS) in the formulated drug solution in units of mg of DMTS per mL of formulated solution:

(1) Injection  Volume  μL=X grat×YmgDMTS/kgratZmgDMTS/mLsolution.

### 2.7. Blood Collection from Catheter Implanted Rats

Syringes, needles, and collecting tubes were sterilized and rinsed with a small volume of heparin. The rat was placed in the rat holder. The plug of the catheter was carefully removed using a pair of tweezers. The lock solution was drawn out using a heparinized syringe until blood appeared in the catheter. The required amount of blood was collected and transferred into to a new heparinized microcentrifuge tube. After collecting blood, the same volume of saline was administered to the rat followed by a 100 *µ*L volume of lock solution injection. Blood was stored at 4°C until use.

### 2.8. Extraction and Analysis of DMTS from Rat Blood Samples

After injecting the DMTS solution (50 mg/mL in 15% Poly80 solution) to the catheter implanted rats, about 1300 *µ*L of blood samples was taken using heparinized syringes, transferred to heparinized microcentrifuge tubes at various predetermined time intervals and kept in ice/refrigerator until use. For the analysis, 1200 *µ*L of blood from each sample was transferred to a heparinized microcentrifuge tube and centrifuged (4°C, 13,500 rpm) for 10 min. The plasma portion was carefully removed using micropipette and discarded. The cells portion was sonicated for 10 min and 400 *µ*L of this sample was extracted with 400 *µ*L of cyclohexanone in a new microcentrifuge tube. To facilitate the partitioning of DMTS, the extraction mixture was vortexed for 30 seconds and shaken on an orbital mixer for 5 min (2045 rpm, 3 mm orbit). To ensure good separation of the organic and aqueous layer, the extraction mixture was centrifuged (4°C, 13,500 rpm) for 10 min. Approximately 40 *μ*L of the upper cyclohexanone layer was transferred into a screw cap HPLC vial containing a Polyspring insert. A 25 *μ*L aliquot drawn from this vial was analyzed by HPLC as described earlier.

### 2.9. Determining Intravascular Residence Time

For the residence time determination, Poly80 formulated DMTS solutions (50 mg/mL 15% Poly80) were injected to the catheter implanted rats at the dose of 20 mg/mL. Blood samples (approximately 1200 *µ*L) were taken at 0, 15, 60, 90, 120, and 240 min after the DMTS injection and analyzed as described earlier. Three rats were used for the study.  Table 1 shows the sampling time and the injection volumes for each rat. Blood samples were taken from rat #1 at 15 and 240 min, from rat #2 at 0 and 60 min, and from rat #3 at 90 and 120 min, after the injection.

## 3. Results and Discussion

### 3.1. Blood Partitioning of DMTS

**Figure 1:**
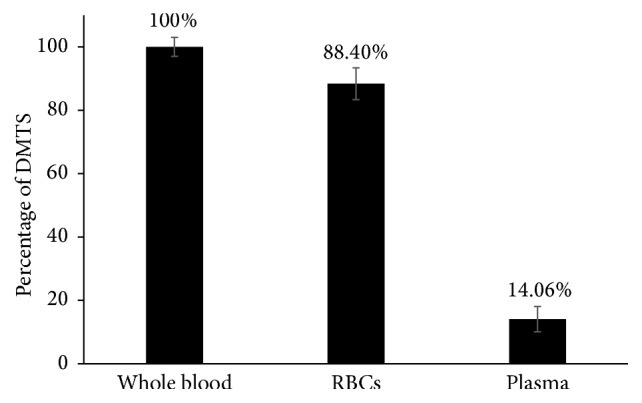
Partitioning of DMTS in blood between plasma and red blood cells (RBCs). Results represent the mean ± SD; *n* = 3.

In modern drug development, drug concentration assays have almost exclusively used plasma as a matrix rather than the whole blood. There are various theories behind this method including assay sensitivity, matrix interference, protein binding, and free drug movement. On the other hand, there are always some arguments about partitioning of hydrophobic molecules, which can be absorbed by, and concentrated in, RBCs [22–24]. Therefore, to determine the matrix for the method, partitioning of DMTS in blood was studied.  Figure 1 clearly reveals the ability of RBCs to absorb DMTS approximately 6.3 times higher than plasma. Therefore, in this research we use RBCs as the matrix to improve the sensitivity.

### 3.2. Analysis of Blood Samples

**Scheme 1:**
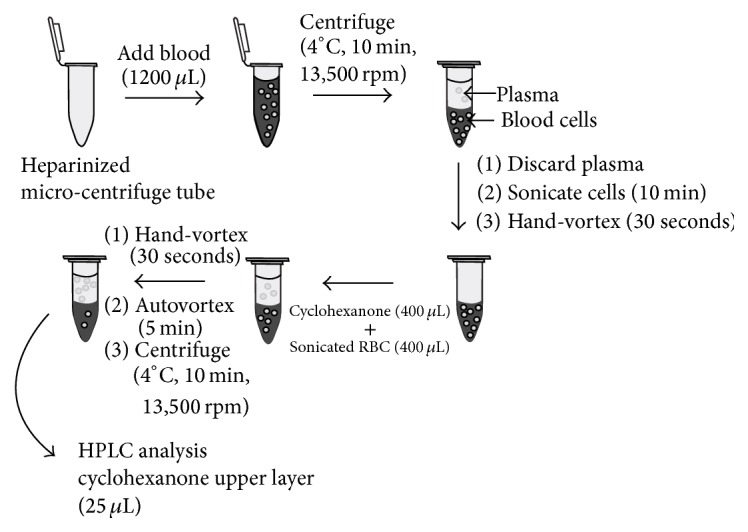
Liquid-Liquid extraction method for determining DMTS in blood.

Scheme 1 summarizes the newly developed liquid-liquid extraction method for determining DMTS from rat blood samples after intravenously administered Poly80 formulated DMTS at the dose of 20 mg/kg. HPLC peaks were identified in the chromatogram when DMTS in cyclohexanone was injected to the HPLC column. Using the previously described HPLC parameters, DMTS showed a retention time of 9.5 min.

**Figure 2:**
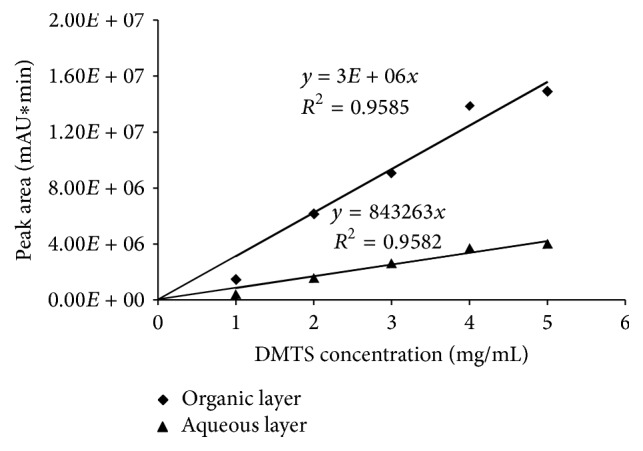
Partitioning of DMTS in 15% Poly80 (aqueous phase) and cyclohexanone (organic layer).

During the method development cyclohexanone as the choice of organic solvent to extract DMTS from the aqueous solution (15% Poly80) showed good partitioning (partition coefficient = 3.7) between the organic and aqueous phases.  Figure 2 shows the partitioning of DMTS between 15% Poly80 as the aqueous phase and cyclohexanone as the organic phase.

The same sample preparation method was used for the calibration curve and the rat blood samples. Despite the good partition with cyclohexanone, in the blood samples the extraction efficiency was relatively low but reproducible. This extraction method provided an effective tool for determining DMTS concentration in rat samples after intravenous administration of Poly80-DMTS for half-life estimation.

### 3.3. Calibration Curve for Analyzing DMTS in Rat Blood

**Figure 3:**
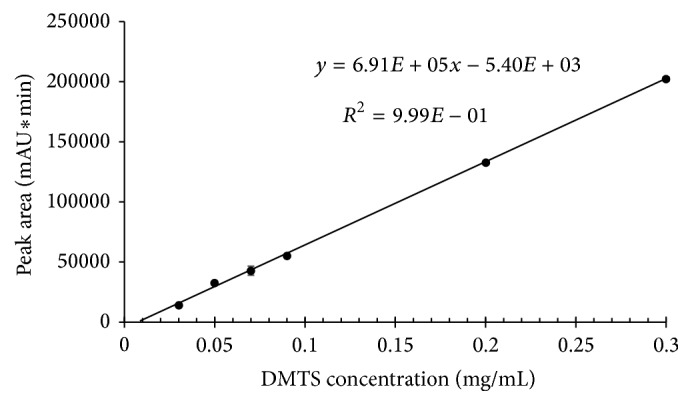
HPLC-UV calibration curve for DMTS after extraction by cyclohexanone from blood. Results represent the mean ± SD; *n* = 2.

Blood samples spiked with DMTS to yield concentrations of 0.01, 0.03, 0.05, 0.07, 0.09, 0.20, and 0.30 mg/mL were extracted as described above and analyzed by HPLC with UV detection. The calibration curve prepared with this data is shown in Figure 3. The lowest point has been omitted from the graph because it was below the quantitation limit.

The signal standard deviation from the calibration line is found to be 2330. Based on this the limit of detection for DMTS via this method is estimated to be 0.010 mg/mL, and the limit of quantitation for DMTS is 0.034 mg/mL. Thus, this calibration curve provides a mechanism for determining DMTS in the range between 0.04 and 0.30 mg/mL. The equation for the calibration curve gained from the cyclohexanone extraction from blood is *y* = 6.91 · 10^5^ *x* − 5.40 · 10^3^ (*R* ^2^ = 0.9994).

### 3.4. Determining Intravascular Residence Time

For the residence time determination Poly80 formulated DMTS (50 mg/mL 15% Poly80) was injected intravenously into rats at the dose of 20 mg/mL, and blood samples were taken at regular time intervals and analyzed as described earlier. Three rats were used for the study.  Table 1 shows the sampling time and the injection volumes for each rat. Blood samples were taken from rat #1 at 15 and 240 min after injection, from rat #2 at 0 and 60 min after injection, and from rat #3 at 90 and 120 min after injection. Blood samples were extracted with cyclohexanone and analyzed with HPLC-UV as described above.

**Figure 4:**
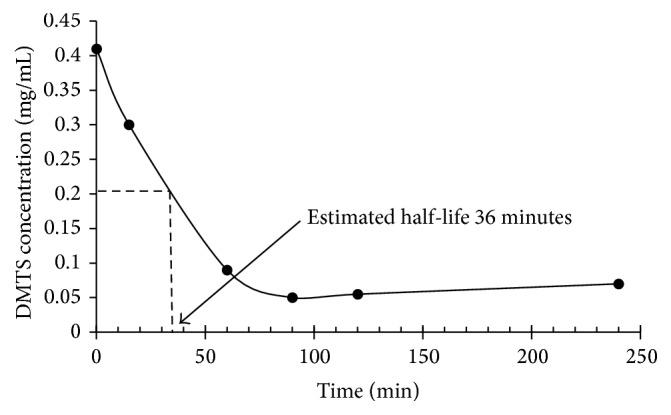
Half-life determination for DMTS-Poly80 graphically from the plotted DMTS concentrations in blood versus sampling times after intravenous injection of DMTS-Poly80 in rat model.

Based on these preliminary results, the half-life of DMTS in blood is estimated to be 36 mins (Figure 4). This circulation time allows providing protection against CN for a reasonable time interval.

## 4. Conclusions

A new HPLC-UV absorbance method for determining the concentration of DMTS in blood has been developed. The method is based on DMTS extraction by cyclohexanone. The range of reliable quantitation for rat blood was found to be 0.04–0.30 mg/mL. This includes the expected DMTS concentration ranges in blood with the determined maximum tolerable dose of DMTS (20 mg/kg intravenously). This analytical method was used for determining the DMTS concentrations in blood after intravenous injection of Poly80-DMTS at the dose of 20 mg/kg and for estimating the half-life graphically. The results of the DMTS intravascular residence time study indicated a circulation half-life of 36 min in a rat model, suggesting that the circulation time of DMTS is long enough to provide a reasonable time interval for CN antagonism.

## Figures and Tables

**Table 1:** Experimental parameters for the DMTS intravascular residence time study.

Experimental parameters	DMTS dose (mg/kg)	Volume of DMTS-Poly80 (50 mg/mL) (*µ*L)	Sampling time after injection (min)
Rat #1	20	142	15 & 240
Rat #2	20	136	0 & 60
Rat #3	20	144	90 & 120
